# Effects of rewarming with extracorporeal membrane oxygenation to restore oxygen transport and organ blood flow after hypothermic cardiac arrest in a porcine model

**DOI:** 10.1038/s41598-021-98044-2

**Published:** 2021-09-23

**Authors:** Jan Harald Nilsen, Torstein Schanche, Sergei Valkov, Rizwan Mohyuddin, Brage Haaheim, Timofei V. Kondratiev, Torvind Næsheim, Gary C. Sieck, Torkjel Tveita

**Affiliations:** 1 Anaesthesia and Critical Care Research Group, Department of Clinical Medicine, UiT The Arctic University of Norway, 9037 Tromsø, Norway; 2 Department of Research and Education, Norwegian Air Ambulance Foundation, 1441 Drøbak, Norway; 3 Division of Surgical Medicine and Intensive Care, University Hospital of North Norway, 9038 Tromsø, Norway; 4 Department of Physiology & Biomedical Engineering, Mayo Clinic, Rochester, MN USA

**Keywords:** Experimental models of disease, Cardiovascular models

## Abstract

We recently documented that cardiopulmonary resuscitation (CPR) generates the same level of cardiac output (CO) and mean arterial pressure (MAP) during both normothermia (38 °C) and hypothermia (27 °C). Furthermore, continuous CPR at 27 °C provides O_2_ delivery (ḊO_2_) to support aerobic metabolism throughout a 3-h period. The aim of the present study was to investigate the effects of extracorporeal membrane oxygenation (ECMO) rewarming to restore ḊO_2_ and organ blood flow after prolonged hypothermic cardiac arrest. Eight male pigs were anesthetized and immersion cooled to 27 °C. After induction of hypothermic cardiac arrest, CPR was started and continued for a 3-h period. Thereafter, the animals were rewarmed with ECMO. Organ blood flow was measured using microspheres. After cooling with spontaneous circulation to 27 °C, MAP and CO were initially reduced to 66 and 44% of baseline, respectively. By 15 min after the onset of CPR, there was a further reduction in MAP and CO to 42 and 25% of baseline, respectively, which remained unchanged throughout the rest of 3-h CPR. During CPR, ḊO_2_ and O_2_ uptake (V̇O_2_) fell to critical low levels, but the simultaneous small increase in lactate and a modest reduction in pH, indicated the presence of maintained aerobic metabolism. Rewarming with ECMO restored MAP, CO, ḊO_2_, and blood flow to the heart and to parts of the brain, whereas flow to kidneys, stomach, liver and spleen remained significantly reduced. CPR for 3-h at 27 °C with sustained lower levels of CO and MAP maintained aerobic metabolism sufficient to support ḊO_2_. Rewarming with ECMO restores blood flow to the heart and brain, and creates a “shockable” cardiac rhythm. Thus, like continuous CPR, ECMO rewarming plays a crucial role in “the chain of survival” when resuscitating victims of hypothermic cardiac arrest.

## Introduction

During the past decades, the overall mortality of accidental hypothermia patients has decreased from 52 to 80% in previous reports^1,2^ to the present 28–35%^3–6^. This favourable outcome is, however, closely linked to accidental hypothermia patients with maintained spontaneous circulation during rescue and rewarming, whereas survival rate of patients in hypothermic cardiac arrest during rescue is much lower. The recommended treatment of hypothermic cardiac arrest is rapid transfer of the patient under continuous cardiopulmonary resuscitation (CPR) to a hospital equipped for in-hospital rewarming using extracorporeal membrane oxygenation (ECMO)^7^.

Case reports of accidental hypothermia patients in hypothermic cardiac arrest, also from our own hospital, have documented survival with favourable neurologic outcome^8–13^. Survival rates without neurologic impairment after ECMO rewarming ranges from 47 to 63% in different studies^8,14–16^. Thus, there is potential to improve treatment of this patient group to further lower their mortality rate. This view finds support by a recent survey reporting no change in survival rate over the last 30 years when using cardiopulmonary bypass (CPB) to rewarm patients from hypothermic cardiac arrest^17^. After extracorporeal rewarming these patients often need cardiopulmonary support, making ECMO the preferred rewarming method^16^, which can be continued for days, if needed.

To improve treatment of accidental hypothermia patients, it is important to systematically investigate effects of all treatment modalities applied during rescue and transport. Optimally, this new knowledge should be collected when analysing data from registries of accidental hypothermia patients, but such registries are relatively new^18^, and the volume of patient data is still limited. Therefore, detailed new information needs to be collected in preclinical animal experiments.

Accordingly, we have established a porcine model of accidental hypothermia and rewarming, in which we recently reported that after 15 min of CPR for ventricular fibrillation at 38 °C vs. 27 °C, hemodynamic function, global O_2_ delivery (ḊO_2_), and organ blood flow were reduced to the same levels irrespective of core temperature^19^. However, during CPR at 38 °C, deterioration of hemodynamic function and organ blood flow occurred within 45 min, whereas at 27 °C, continued CPR for up to 3-h provided adequate ḊO_2_ to support aerobic metabolism in critical organs^19^. This suggests that global V̇O_2_ is higher at 38 °C, and that CPR provides insufficient ḊO_2_ to maintain aerobic metabolism at this temperature.

Although our model of hypothermic cardiac arrest may benefit from hypothermia-induced reduction of metabolism during CPR, the prolonged hypothermic low-flow condition followed by reperfusion may impair organ function^20^. Thus, it remains to be determined if ECMO rewarming will lead to restitution of function in vital organs. Accordingly, the aim of the present study was to evaluate if spontaneous cardiac activity and ḊO_2_ to critical organs can be re-established during ECMO rewarming following 3-h of continuous CPR at 27 °C in a porcine model.

## Results

All animals had spontaneous circulation during cooling to 27 °C. Hemodynamic variables and the electrocardiogram were continuously monitored throughout the experiment.

### Immersion cooling and 3-h CPR at 27 °C

#### Hemodynamics (Fig. 1A,B)

**Figure 1:**
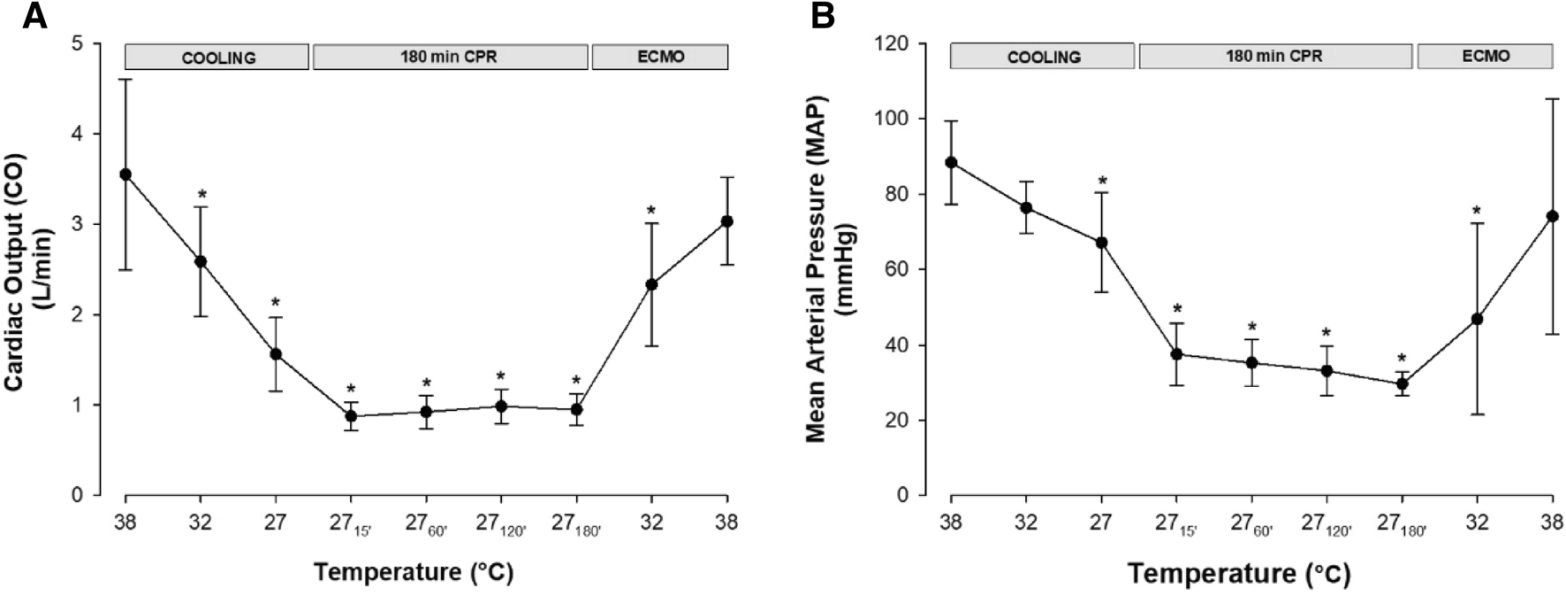
Measurements of hemodynamic function during cooling, 3-h CPR at 27 °C, and ECMO rewarming. (**A**) Cardiac output. (**B**) Mean arterial pressure. n = 8, values are mean ± SD. *p < 0.05 statistically significantly different from baseline value.

All statistical comparisons are made in reference to individual baseline (38 °C) values. Cooling reduced MAP significantly from 88 ± 11 to 76 ± 7 mmHg at 27 °C (− 24%). After 15 min of CPR, MAP fell to 38 ± 8 mmHg (− 58%), and remained at this reduced level throughout the remaining 3-h period of CPR. Similarly, after cooling to 27 °C, CO fell significantly from 3.6 ± 1.1 to 1.2 ± 0.4 l/min (− 56%). After 15 min of CPR, CO fell even further to 0.9 ± 0.2 l/min (− 75%) and remained at this reduced level during the remaining 3-h period of CPR.

#### O_2_ transport and extraction (Fig. 2A,C)

**Figure 2:**
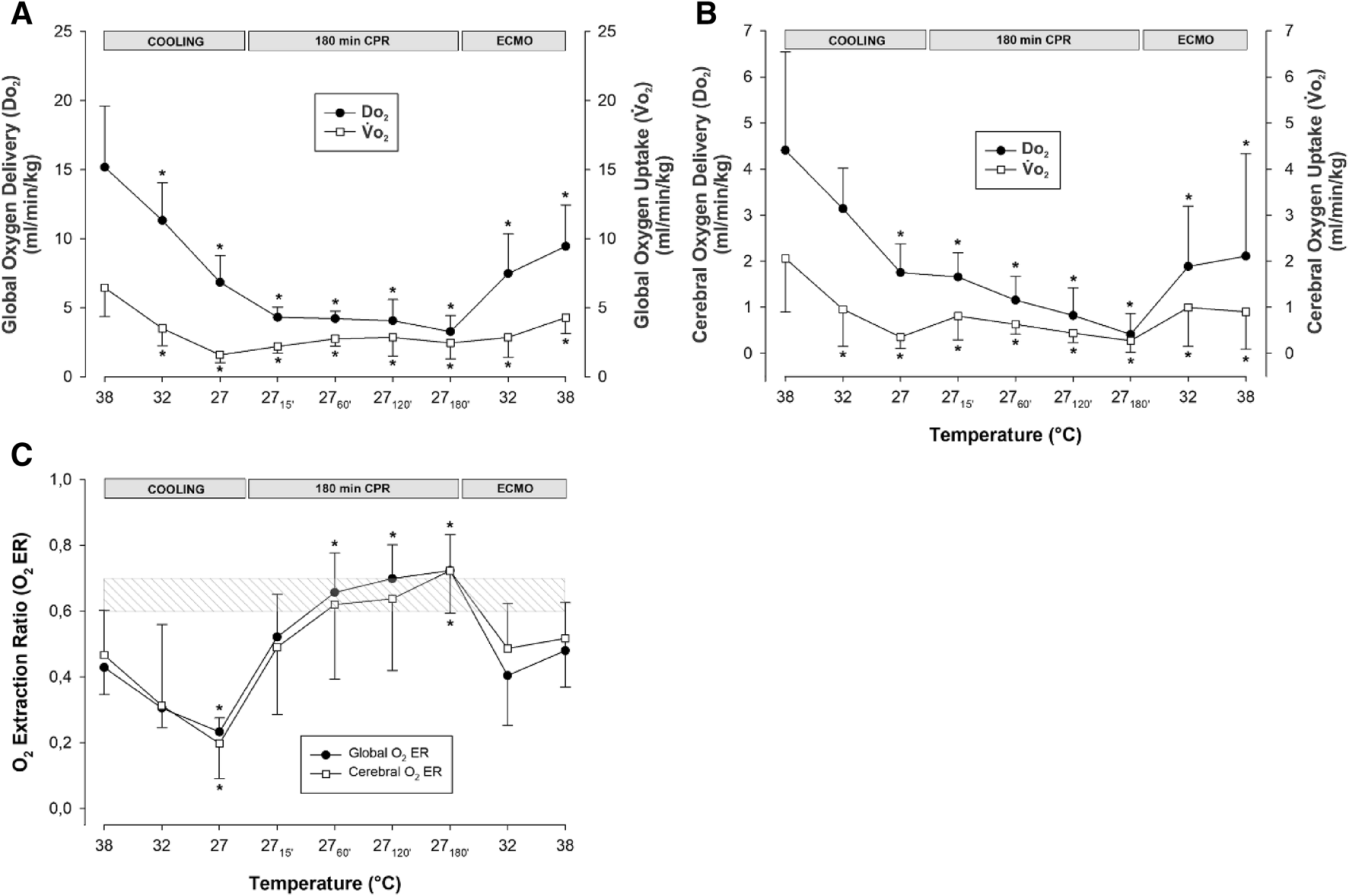
Global and cerebral oxygen delivery, uptake and oxygen extraction ratio. (**A**) Global oxygen delivery (ḊO_2_), and global oxygen uptake (V̇O_2_). (**B**) Cerebral ḊO_2_, and cerebral V̇O_2_. (**C**) Global and cerebral oxygen extraction ratio. n = 8, values are mean ± SD. *p < 0.05 statistically significantly different from baseline value. Striated area indicates critical level of extraction ratio.

Global ḊO_2_ was reduced significantly during cooling to 27 °C from 15.2 ± 4.4 to 6.8 ± 1.9 ml/min/kg (− 55%). Similarly, V̇O_2_ decreased during cooling to 27 °C from 6.8 ± 2.5 to 1.7 ± 0.6 ml/min/kg (− 75%). After 15 min of CPR, ḊO_2_ was further reduced to 4.3 ± 0.7 (− 72%), and V̇O_2_ was reduced to 2.2 ± 0.5 (− 68%) ml/min/kg, and both ḊO_2_ and V̇O_2_ remained at these reduced levels throughout the remaining 3-h period of CPR. Global O_2_ extraction ratio (V̇O_2_/ḊO_2_) was significantly reduced by cooling to 27 °C, and after 60 min of CPR, extraction ratio reached 0.68 ± 0.11, the reported critical extraction ratio necessary to provide aerobic metabolism^21^. Due to the stable CO throughout the 3-h period of CPR period, extraction ratio remained at this elevated level.

#### Arterial lactate, pH, and central venous O_2_ saturation (SvO_2_) (Table 1)

**Table 1:** Plasma biochemical variables, and values for cerebral pressures.

	38 °C	27 °C	27 °C_15 min_	27 °C_3-h_	RW 32 °C	RW 38 °C
pH	7.55 ± 0.05	7.42 ± 0.03*	7.4 ± 0.02*	7.20 ± 0.08*	7.27 ± 0.11*	7.39 ± 0.1*
Hb (g/dl)	8.2 ± 1.1	8.7 ± 1.2	9.2 ± 1.3	7.9 ± 0.9	5.5 ± 1.3*	5.3 ± 1.6*
Hct (%)	27 ± 2	27 ± 4	29 ± 4	25 ± 4	16 ± 4*	17 ± 5*
Lactate (mmol/l)	1.0 ± 0.7	0.5 ± 0.1	0.9 ± 0.3	5.2 ± 2.0	5.8 ± 2.6*	5.1 ± 2.6
BE (mmol/l)	5.6 ± 2.7	3.3 ± 3.5	2.2 ± 1.6*	− 6.3 ± 2.4*	− 8.9 ± 4.0*	− 8.2 ± 3.3*
HCO_3_^−^ (mmol/l)	30 ± 3	27 ± 3	26 ± 1*	18 ± 2*	20 ± 2*	20 ± 3*
K^+^ (mmol/l)	3.3 ± 0.4	2.6 ± 0.4	3.1 ± 0.3	5.3 ± 1.5*	4.5 ± 0.4	4.5 ± 0.7
PaO_2_ (kPa)	13 ± 5	16 ± 4	46 ± 28*	15 ± 13	73 ± 3*	66 ± 2*
PaCO_2_ (kPa)	4.4 ± 0.3	5.8 ± 0.8	5.8 ± 0.6	7.5 ± 1.5*	5.2 ± 1.9	3.7 ± 0.9
SaO_2_ (%)	99 ± 2	99 ± 1	100 ± 0	82 ± 26	100 ± 0	100 ± 0
SvO_2_ (%)	60 ± 11	78 ± 4	52 ± 15	21 ± 8*	68 ± 17	61 ± 17
SvO_2_ jug.bulb (%)	58 ± 12	85 ± 13*	66 ± 21	26 ± 12*	59 ± 18	66 ± 12
CVP (mmHg)	6 ± 1	5 ± 2	19 ± 17*	14 ± 7	13 ± 6	15 ± 4
ICP (mmHg)	14 ± 3	13 ± 6	21 ± 5*	17 ± 4	17 ± 4	22 ± 7*
CPP (mmHg)	74 ± 12	55 ± 11	17 ± 9*	13 ± 5*	32 ± 21*	52 ± 32

Hct, haematocrit; BE, base excess; HCO_3_^−^, bicarbonate; P_a_O_2_, arterial partial O_2_ pressure; P_a_CO_2_, venous partial O_2_ pressure; Sat_a_O_2_, arterial O_2_ saturation; Sat_v_O_2_, venous O_2_ saturation; CVP, central venous pressure; ICP, intra-cerebral pressure; CPP, cerebral perfusion pressure (CPP = MAP − ICP). n = 8, values are mean and SD. *p < 0.05 statistically significantly different from baseline value.

Cooling to 27 °C and the 3-h period of CPR caused an almost linear reduction in pH from 7.55 ± 0.05 to 7.20 ± 0.08, simultaneously with an increase in serum lactate from 0.98 ± 0.68 to 5.18 ± 1.96 mmol/l. These changes in pH and lactate took place while SvO_2_ fell from 57 ± 12 to 21 ± 5%.

#### Organ blood flow (Fig. 3A–E, Table 2)

**Figure 3:**
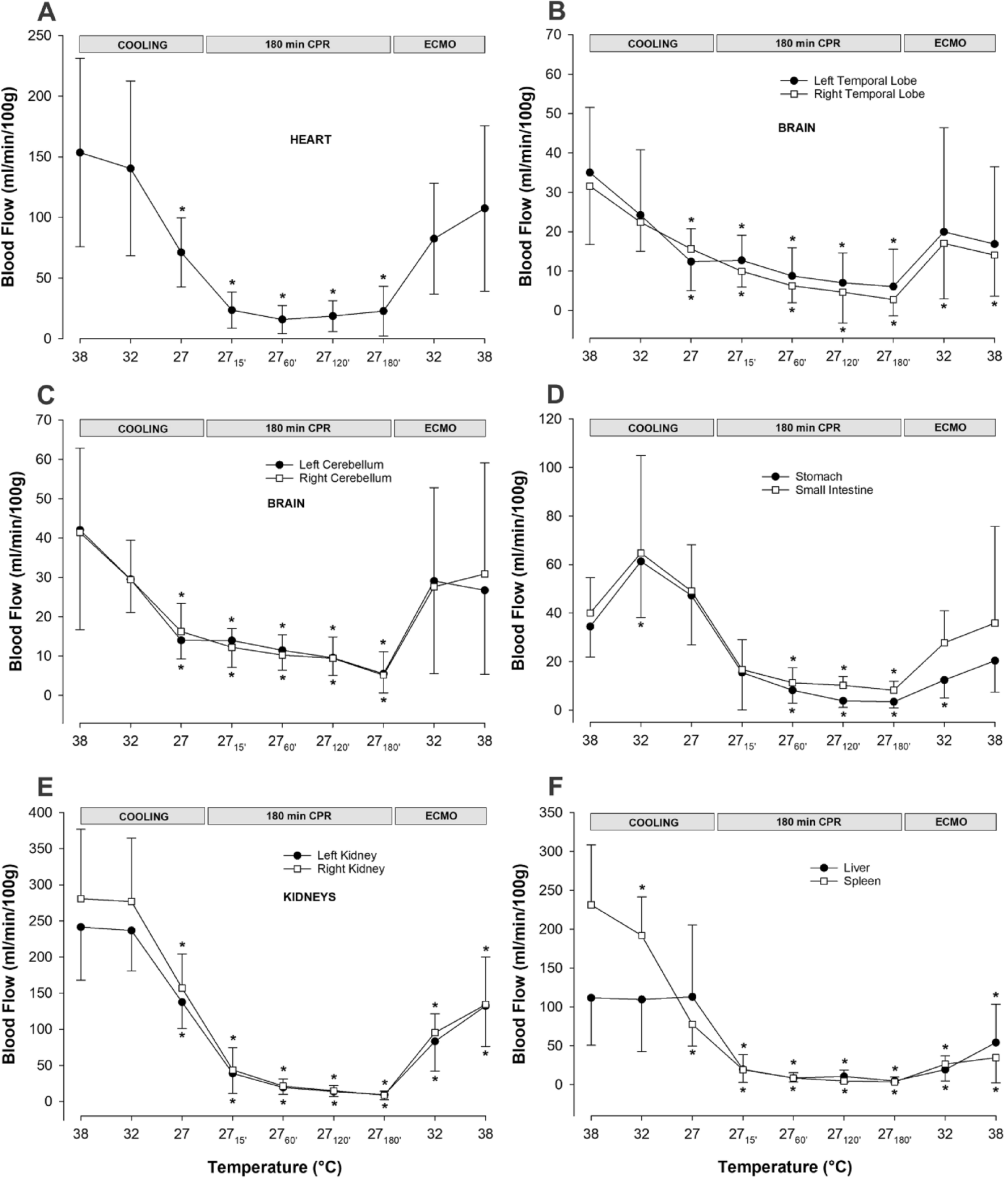
Regional blood flow during cooling, 3-h CPR at 27 °C, and ECMO rewarming. (**A**) Myocardial blood flow. (**B**) Blood flow in left and right temporal lobes. (**C**) Blood flow in left and right cerebellar hemispheres. (**D**) Blood flow in stomach and small intestine. (**E**) Renal blood flow. (**F**) Blood flow in liver and spleen. n = 8, values are mean ± SD. *p < 0.05 statistically significantly different from baseline value.

**Table 2:** Organ blood flow (ml/min/100 g).

	38 °C	27 °C	27 °C_15 min_	27 °C_3-h_	RW 32 °C	RW 38 °C
Heart	153 ± 78	71 ± 28*	24 ± 15*	23 ± 20*	82 ± 46	107 ± 68
Left temp. lobe	35 ± 17	12 ± 7*	12 ± 6*	6 ± 9*	20 ± 26	17 ± 20
Right temp. lobe	32 ± 15	16 ± 5*	10 ± 4	3 ± 4*	17 ± 14*	14 ± 10*
Left cerebellum	42 ± 21	14 ± 5*	14 ± 3*	6 ± 6*	29 ± 24	27 ± 21
Right cerebellum	41 ± 24	16 ± 7*	12 ± 5*	5 ± 4*	27 ± 22	31 ± 28
Left kidney	242 ± 74	138 ± 37*	39 ± 28*	9 ± 7*	83 ± 41*	132 ± 56*
Right kidney	281 ± 96	157 ± 47*	44 ± 31*	9 ± 5*	95 ± 26*	134 ± 66*
Liver	112 ± 61	113 ± 92	20 ± 19*	5 ± 5*	19 ± 14*	54 ± 49*
Stomach	34 ± 13	47 ± 20	15 ± 15	3 ± 3*	12 ± 7*	20 ± 13
Small intestine	40 ± 15	49 ± 19	17 ± 12	8 ± 4*	28 ± 13	36 ± 40
Spleen	231 ± 77	78 ± 28*	19 ± 16*	4 ± 1*	26 ± 11*	35 ± 32*

n = 8, values are mean and SD. *p < 0.05 statistically significantly different from baseline value.

Compared to baseline, myocardial blood flow (Fig. 3A) was significantly reduced (− 54%) after cooling to 27 °C. After ventricular fibrillation and 15 min CPR, myocardial blood flow was further reduced (− 85%), and remained at this reduced level during the remaining 3-h period of CPR. After cooling to 27 °C, blood flow in the temporal lobes (Fig. 3B) was significantly reduced (left lobe − 65%, and right lobe − 51%). After 15 min of CPR, there was a further reduction in blood flow to the left and right temporal lobe, − 64% and − 68%, respectively, and after 3-h of CPR, blood flow to the left and the right temporal lobes were reduced to − 83% and − 91% of baseline, respectively. Compared to baseline at 38 °C, cooling to 27 °C significantly reduced blood flow to the left (− 67%) and right (− 61%) cerebellar hemispheres (Fig. 3C), but CPR for 15 min did not lead to any further reduction in blood flow. However, after 3-h of CPR, blood flow to the left (− 87%) and the right (− 88%) cerebellar hemispheres was further reduced. Abdominal organs showed a varying reductions in blood flow during cooling, as well as during 3-h period of CPR (Fig. 3D–F). After cooling to 27 °C, blood flow to the stomach and small intestine increased by + 38% and + 23%, respectively (Fig. 3D). However, after 3-h of CPR, blood flow to the stomach and small intestine was severely reduced by − 90% and − 79% of baseline, respectively. Cooling to 27 °C significantly reduced blood flow to the right (− 44%) and left (− 43%) kidneys (Fig. 3E), and renal blood flow was almost completely shut off after 3-h of CPR (− 97%, and − 96%, respectively). Liver blood flow was unaltered after cooling to 27 °C, whereas blood flow to the spleen was reduced (− 66%) (Fig. 3F). Both organs had severely impaired blood flow during CPR, and after 3-h, blood flow was reduced by − 97% in the liver and − 99% in the spleen compared to baseline at 38 °C.

### ECMO rewarming

The mean time required to rewarm blood temperature to 32 °C was 17 min with an additional 45 min to rewarm blood temperature to 38 °C. Ringer acetate was added to the ECMO circuit to maintain venous access pressure above − 100 mmHg.

#### Return of cardiac rhythm

All 8 pigs achieved sinus rhythm on ECMO, one spontaneously at 29 °C, two after cardioversion at 32 °C, and two after cardioversion at 38 °C. Three animals resumed a sinus rhythm only after sternotomy and pericardiotomy, to evacuate blood congesting in the mediastinum and in pericardium, before internal cardioversion at 38 °C.

#### Blood flow, pressure, and rewarming rate during ECMO

In the ECMO circuit, pump flow rate and MAP (Fig. 1A,B) were adjusted to mimic hemodynamic characteristics of the individual animal during cooling. Statistical comparisons were based on comparisons to individual baseline (38 °C) values. At 27 °C, the circuit was started at a flow of 1 l/min, gradually increased to 2.0–2.5 l/min at 32 °C, and finally 3.0–3.5 l/min at 38 °C. No vasoactive pharmacologic intervention was required to adjust pressure generation during rewarming. In each experiment, total volume of Ringer acetate added varied between 2000 to 6000 ml (mean 3750 ml).

#### O_2_ transport and extraction (Fig. 2A–C)

Global ḊO_2_ increased during ECMO rewarming but at 38 °C, it was still significantly reduced compared to baseline (15.2 ± 4.4 vs. 9.4 ± 3.0 ml/min/100 g; − 38%). Similarly, global V̇O_2_ increased during rewarming, but at 38 °C, it was still reduced compared to baseline (6.8 ± 2.5 vs. 4.3 ± 1.1 ml/min/100 g; − 34%).

During rewarming, cerebral ḊO_2_ and V̇O_2_ (Fig. 2B) increased, but at 38 °C, they were both still reduced compared to baseline (cerebral ḊO_2_: 4.41 ± 2.14 vs. 2.11 ± 2.23; − 50% and V̇O_2_: 2.08 ± 1.17 vs. 0.85 ± 0.87 ml/min/100 g; − 56%). Both global and cerebral extraction ratio (Fig. 2C) fell to values < 0.7 during rewarming but at 38 °C, they were unchanged compared to baseline values.

After rewarming arterial pH levels (Table 1) returned to baseline, 7.39 ± 0.1 vs. 7.55 ± 0.05. Serum lactate levels (Table 1) were highest at 32 °C (5.8 ± 2.59), fell during rewarming to 38 °C, but were still significantly elevated compared to baseline (5.1 ± 2.62 vs. 0.98 ± 0.68 mmol/l). After rewarming SvO_2_ (Table 1) was restored to 60%.

#### Organ blood flow (Fig. 3A–E, Table 2)

Rewarming elevated blood flow to all organs, but statistical analyses showed that blood flow returned to baseline in only a few organs. Myocardial blood flow was restored after rewarming. Similarly, blood flow to the left temporal lobe was restored, whereas blood flow to the right temporal lobe was still significantly reduced (− 56%). Rewarming restored blood flow to both cerebellar hemispheres. In most abdominal organs, rewarming only led to partial restoration of blood flow. Blood flow to the stomach and small intestine was restored, whereas, compared to baseline, blood flow remained significantly reduced in the liver (− 52%) and spleen (− 82%). Compared to baseline 38 °C, renal blood flow was significantly reduced after rewarming in both the left (− 45%), and right (− 53%) kidney.

### Plasma biomarkers (Table 3)

**Table 3:** Serum biomarkers for organ function and organ injury.

Organ	38 °C	27 °C	27 °C_3-h_	RW 38 °C
**Brain**				
S100β (pg/ml)	64 ± 44	40 ± 28	85 ± 56	89 ± 53
UCHL1 (pg/ml)	91 ± 12	108 ± 48	92 ± 9	95 ± 15
GFAP (pg/ml)	8.8 ± 5.6	8.4 ± 5.1	9.8 ± 4.9	14.7 ± 8.0*
NSE (ng/ml)	–	–	–	–
**Heart**				
CK-MB (ng/ml)	1.7 ± 1.9	2.0 ± 2.1	1.0 ± 0.8	1.2 ± 1.0
Troponin T (pg/ml)	65.8 ± 51.2	54.1 ± 47.2	47.4 ± 39*	49.2 ± 46.5*
**Kidney**				
Carbamide (mmol/l)	1.6 ± 0.3	2.0 ± 0.5	2.4 ± 0.6*	2.2 ± 0.7*
Creatinine (μmol/l)	57.1 ± 7.8	49.3 ± 11.3*	61.6 ± 12.5	47.4 ± 9.9*
Activin-A (pg/ml)	–	–	–	–
**Liver/pancreas**				
ASAT (U/l)	40.6 ± 5.3	51.4 ± 9.6	264.0 ± 81.6*	374.1 ± 226.5*
ALAT (U/l)	72.6 ± 16.8	67.2 ± 15.1*	70.4 ± 12.6	55.7 ± 12.1*
γ-GT (U/l)	31.1 ± 9.4	27.1 ± 6.8	22.8 ± 4.8*	15.4 ± 5.2*
Amylase (U/l)	1744 ± 562	1591 ± 478	1359 ± 466*	879 ± 352*
Lipase (U/l)	–	–	–	–
Bilirubin (μmol/l)	–	–	–	–
ALP (U/l)	–	–	–	–

UCHL1, Ubiquitin Carboxyl-terminal Esterase-L1; GFAP, Glial Fibrillary Acidic Protein; NSE, Neuron-Specific Enolase; CK-MB, Creatine Kinase-Muscle/Brain isozyme; ASAT, Aspartate Aminotransferase; ALAT, Alanine; Aminotransferase; γ-GT, γ-Glutamyl Transpeptidase; ALP, Alkaline Phosphatase. n = 8, values are mean and SD. *p < 0.05 statistically significantly different from baseline value.—indicate serum level below lower level of detection.

Significant (six–tenfold) increase in ASAT appeared after 3-h CPR and remained after rewarming. Also level of glial fibrillary acidic protein (GFAP) was increased after rewarming.

## Discussion

This experiment demonstrates that following 3-h of CPR for hypothermic cardiac arrest at 27 °C, ḊO_2_ and organ blood perfusion were reduced but that ECMO rewarming provided blood flow, MAP, and ḊO_2_ to support global aerobic organ metabolism. After rewarming to 38 °C, organ blood flow was unequally restored, but with an apparent preference to the brain and heart, indicating the patency of autonomic blood flow regulation to support O_2_ delivery to critical organs. Furthermore, 3-h of continuous CPR at 27 °C maintained MAP, CO, and organ blood flow at the same reduced level, with adequate ḊO_2_ to enable aerobic metabolism. In this respect, the findings of the present study are consistent with those of a previous study^19^. The reason for testing 3-h CPR is that our hospital is located in a scarcely populated catchment area, above the Arctic Circle, making evacuation and transportation time with air ambulance typically 3–4 h for patients with hypothermic cardiac arrest in need of in-hospital rewarming.

### Resuscitation during hypothermia and normothermia

The results of the present study confirm that after ECMO rewarming, prognostic outcome markers are favourable as compared to survivors of normothermic cardiac arrest^22^. These markers include higher pH, low level of plasma lactate, and a shockable cardiac rhythm. In fact, hypothermia may provide protective effects during cardiac arrest that would mitigate the complex pathophysiologic processes created by the prolonged low-flow condition. Protective mechanisms at low temperatures relate to the general slowing of enzymatic activities, particularly those that are ATP-dependent. On the other hand, these protective effects may be partly offset by harmful effects of hypothermic exposure, which, even in the absence of ischemia or hypoxia may cause end-organ dysfunction. We have previously documented hypothermia-rewarming induced cardiac dysfunction in both in vivo and in vitro models^23–28^. Underlying pathophysiologic mechanisms include derangement in metabolism and calcium homeostasis^23–25,29^, elevated protein kinase A levels with increased phosphorylation in myocardial contractile proteins^27,28,30^, and reactive oxygen species formation^31^. Likewise, after rewarming, we have documented derangements in renal^32^ and nervous tissue morphology^33^. Severity of these different pathophysiologic elements are closely related to duration and level of the hypothermic exposure with similarities to what takes place during normothermic low flow ischemia.

### Reperfusion

Rewarming from hypothermia and reperfusion after ischemia share the same treatment strategy; to restore blood flow at the macro-vascular level in an attempt to optimize blood flow at the micro-vascular level to minimize end organ dysfunction. However, alterations in micro-vascular function frequently occur in critically ill patients and with clear implications to development of organ failure. Although reperfusion is the ultimate constituent when resuscitating from periods of ischemia or limited blood flow, as following prolonged hypothermic cardiac arrest, we still have limited knowledge about how the different organs respond to reperfusion. However, all reperfused organs are exposed to complex pathophysiologic processes causing uneven alterations in organ function, collectively termed the post-cardiac arrest syndrome^20^. In addition, considerable variability in metabolic responses in critical organs was recently reported to take place after 30 min of cardiac arrest, as well as after CPB, in an experimental model of cardiac arrest during normothermia^34^.

### Restitution of organ blood flow after rewarming

In the present experiment ECMO rewarming restored MAP and CO in parallel with a return to well below critical value for extraction ratio (0.6–0.7)^21^, return of SvO_2_, whereas global V̇O_2_ remained reduced. The reduced global V̇O_2_ may well be a mirror image of reduced organ function as heterogeneity in the recovery of organ blood flow was evident in most organs investigated.

The brain is the organ most sensitive to ischemic injury and is therefore the limiting organ for survival after cardiac arrest in general^35^. We observe that cerebral ḊO_2_ and V̇O_2_ were both significantly reduced in parallel with a reduction in extraction ratio to far below critical levels. Also, if we compare to human data after cardiac arrest, a patent autoregulation of cerebral blood flow after reperfusion is suggested if decreased cerebral blood flow is matched to decreased V̇O_2_^36^. Deliberate hypothermic cardiac arrest is used for repair of complex cardiovascular conditions, and for cerebral protection the safe use of cardiac arrest for up to 60 min at 8–13 °C^37–39^ has been documented. In the present experiment biomarkers of brain injury disclose no pathological changes as GFAP and UCHL1 (Table 3), both highly selective for CNS injury^40^, are within normal control levels in pigs^41^.

Myocardial blood flow was normalized after the return of spontaneous electro-mechanic activity despite that external heart work was reduced as global circulation was provided by the ECMO circuit indicating the occurrence of reactive hyperaemia. Biomarkers of cardiac injury, CK-MB and Troponin T, were both within normal levels. The significant increase in ASAT is most probably caused by trauma of the thoracic muscles from the automated compression devise.

Reduced renal blood flow appears to be a consequence of the well-documented physiologic mechanisms that compensates for a sudden drop in MAP and/or CO as during cardiac arrest. The biomarker activin-A, also reported to be increased during acute renal failure, was beyond detection levels in our experiment. Reduced blood flow to the spleen can be observed after circulatory shock secondary to emptying stored erythrocytes into the blood stream as a compensatory mechanism^42^. However, the immediate restoration of blood flow to the small intestine and stomach, while liver blood flow was significantly reduced, is more difficult to interpret.

### Adequacy of extracorporeal rewarming for macro- and micro-vessel reperfusion

The recommended treatment for hypothermic cardiac arrest patients is rapid transfer under continuous CPR to a hospital capable of rewarming by use of extracorporeal circulation^7^. The safe use of extracorporeal circulation, routinely applied as CPB during cardiac surgery, is supported by extensive preclinical and clinical research over the past 60 years. This would suggest that extracorporeal circulation/CPB would also be safe for rewarming accidental hypothermia patients. However, a comprehensive preclinical study is lacking. Obvious differences between cardiac surgery patients and accidental hypothermia patients include the way cooling takes place, duration of the hypothermic insult, and patency of O_2_ transport during the insult, factors that also may warrant different approaches for the use of extracorporeal circulation for rewarming.

Restitution of capillary flow is a key element when rewarming accidental hypothermia patients with extracorporeal circulation. Even after exposure to short-term hypothermia with maintained spontaneous circulation, intravascular erythrocyte aggregation has been reported^43^, and other studies have documented that the size of intravascular erythrocyte aggregates during hypothermia was inversely related to blood flow^44^. These changes create a heterogeneous micro-vascular blood flow with perfused capillaries in close vicinity to non-perfused capillaries, which subsequently may cause alterations in tissue O_2_ transport and hypoxia in organs despite restitution of global O_2_ transport. In our effort to restitute systemic hemodynamic function, the micro-vascular hemodynamic function may suffer, a fact that underlines the existence of an uncoupling between macro and micro-vascular circulation^45^. Another important factor to compromise capillary integrity during hypothermia with spontaneous circulation is that increased extravasation of plasma from the intravascular to the interstitial space^46,47^ regularly takes place, and this extravasation is substantially increased when applying extracorporeal circulation for rewarming^47^. Extracorporeal circulation has evolved to become the method of choice for rewarming patients with hypothermic cardiac arrest. However, a recent review has documented impaired micro-vascular integrity as a consequence of CPB during cardiac surgery^48^. By use of sublingual micro-circulatory measurements, numerous reports have documented impaired micro-circulatory perfusion with subsequent reduction of functional capillary density, and these changes may last 24 h after CPB^48^. The reduction in functional capillary density after CPB shear great similarities with those taking place during hypothermia with spontaneous circulation mentioned above.

Taken together, this information points at alterations in capillary integrity and microcirculatory function taking place during accidental hypothermia, which may be prolonged and even aggravated by adding extracorporeal circulation for rewarming. Therefore, future aim must be to establish a refined extracorporeal circulation system for rewarming, using CPB or ECMO, which has the ability to support micro-vascular integrity rather than prolong micro-vascular dysfunction. Based on promising clinical reports^16^ the use of ECMO for rewarming from accidental hypothermia has been recommended as ECMO can also be continued after rewarming for cardio/respiratory support for days, if needed^16,49^.

### Limitations

Perhaps as a consequence of using an automated chest compression device designed for human CPR^50^, the use in our pig model resulted in multiple costa and sternum fractures in all animals. Furthermore, as a consequence of these fractures, blood congesting in the mediastinum and in pericardium necessitated surgical evacuation to manage cardioversion in three out of eight animals during rewarming. This may well be due to the prolonged 3-h period of CPR, although fatal injuries in human patients have not been documented^51^ after conventional CPR using a compression device^52^. To determine organ blood flow, microspheres were injected into the left ventricle during cooling and CPR, but injected directly into a port on the arterial cannula during ECMO rewarming. This may have altered the way microspheres were introduced into the circulation, which may have had impact on blood flow measurements. It would have been of interest to compare effects of ECMO to restore blood flow and ḊO_2_ with a control group rewarmed with conventional methods. However, in pilot studies, when rewarming with other methods than extracorporeal circulation, we were unable to restore cardiac electro-mechanical activity and achieve return of spontaneous circulation after 3-h of CPR at 27 °C. This may, however, be interpreted to support the use of extracorporeal circulation to rewarm patients with hypothermic cardiac arrest.

## Conclusions

This study shows that ECMO rewarming following 3-h of CPR at 27 °C restores hemodynamics and partially or fully re-establishes blood flow to critical organs. Our results showing normal pH albeit elevated lactate levels, absence of hyperkaliaemia, and restored cardiac electro-mechanical activity, support the conclusion that aerobic metabolism is at least partially sustained during hypothermic CPR and ECMO rewarming. Based on these findings, it is pertinent to advocate for continued prehospital CPR during transport of victims of severe accidental hypothermia to centres equipped for rewarming using extracorporeal circulation. Clinical reports favour the use of ECMO rewarming, but extensive research work is needed to optimize the use of extracorporeal circulation techniques for the rewarming of accidental hypothermia patients.

## Materials

### Animals

This study on eight male pigs (24.5–33.0 kg) from a Norwegian stock (Noroc) was approved by the National Animal Research Authority (ref. number: 14/56323) and conform to the guidelines from Directive 2010/63/EU of the European Parliament on the protection of animals used for scientific purposes. The animals, which received humane treatment in accordance with The Norwegian Animal Welfare Act, were penned for 3–5 days in our animal facility after arrival, fed twice daily and had free access to water at all times. The study was carried out in compliance with the ARRIVE guidelines.

### Anaesthesia and instrumentation

The methods for hemodynamic monitoring, immersion cooling, and organ blood flow measurements in the porcine model have been previously reported in detail^53^. Briefly, the animals were fasted overnight and premedication was administered in the pen by an intramuscular bolus of ketamine 20 mg/kg, midazolam 30 mg and atropine 1 mg. After transfer to the research lab, venous access was established in an ear-vein and anaesthesia was induced by an intravenous bolus of fentanyl 10 µg/kg and pentobarbital sodium 10 mg/kg. Continuous anaesthesia was established with intravenous infusion of fentanyl 20 µg/kg/h, midazolam 0.3 µg/kg/h and pentobarbital sodium 4 mg/kg/h. Anaesthesia was discontinued when core body temperature reached 27 °C and re-introduced during the rewarming period.

After induction of anaesthesia, a primary tracheostomy was performed to secure the airway, and following intubation, the animals were ventilated with a positive end-expiratory pressure of 0 cm H_2_0 (Siemens Servo 900D, Solna, Sweden). During ventilation, FIO_2_ was adjusted to maintain PaO_2_ > 10 kPa and alveolar ventilation was adjusted to maintain PaCO_2_ at 4.5–6 kPa uncorrected for temperature (α-stat). During ventricular fibrillation and CPR, FIO_2_ was set to 1.0.

All vascular catheters were placed under ultrasound guidance. An 8-French sheath (Edwards Lifesciences, Irvine, CA, USA) was placed into the left femoral vein to ensure rapid establishment of continuous intravenous anaesthesia. A 7.5-French thermodilution catheter (Edwards Lifesciences, Irvine, CA, USA) was inserted through the sheath in the left femoral vein and advanced to the pulmonary artery. An 8-French Super Arrowflex (Arrow international Inc., Reading, PA, USA) sheath was placed in the left femoral artery and a 7.5-French catheter (Edwards Lifesciences, Irvine, CA, USA) was introduced into the aortic arch. A 10-French Super Arrowflex sheath (Arrow international Inc., Reading, USA) was placed in the right carotid artery, and a 6-French pigtail catheter (Cordis Corporation, Miami, FL, USA) was introduced into the left ventricle of the heart. A 3 mm flow probe (Cardiomed AS, Norway) was placed on the left carotid artery. An 18-gauge central venous catheter (Arrow international Inc., Reading, PA, USA) was placed retrograde into the left jugular bulb. Three 6-French sheaths (Cordis Corporation, Miami, FL, USA) were placed in the right jugular vein, right femoral vein and right femoral artery, respectively to allow for placement of ECMO cannulas during the last 30 min of CPR. A 3.5-French pressure catheter (SPR-524, Millar Instruments Inc., Houston, TX, USA) was introduced into the left hemisphere of the brain through a burr-hole in the skull. A 14-French urinary catheter was introduced into the bladder through a small incision in the abdomen. After instrumentation, a single dose of 5000 IU Heparin was given. The animals were allowed to stabilize for 45 min before starting the experimental protocol.

### ECMO circuit

The ECMO circuit was built with 3/8ʺ tubes for the main circuit and 1/4ʺ tubing for the cannulas. Dual venous tubes were used to provide venous blood for the oxygenator and heat/exchanger (Quadrox-I Adult, Maquet Cardiopulmonary AG, Hirrlingen, Germany), and the oxygenated blood was then pumped into the artery by a centrifugal pump (Rotaflow, Maquet Cardiopulmonary AG, Hirrlingen, Germany). The ECMO circuit was primed with 1000 ml ± 100 ml Ringer’s lactate solution. Correct position of all guidewires and cannulas were verified by x-ray. The 6-French sheaths were used as ports to ensure rapid percutaneous placement of the ECMO cannulas. First a guidewire was inserted through the sheath, then the 6-French sheath was removed, and the flexible guidewire was replaced with a rigid guidewire (Amplatz super-stiff, Boston Scientific, Marlborough, MA, USA) via a guiding sheath. The vessels were then dilated in several steps up to 16-French. This was done on the right jugular vein, the right femoral vein and the right femoral artery. A 15-French × 18 cm ECMO cannula (Bio-Medicus, Medtronic Inc., Minneapolis, MN, USA) was inserted into the right jugular vein, and another into the right femoral artery. A 15-French × 50 cm venous ECMO cannula (Bio-Medicus, Medtronic Inc., Minneapolis, MN, USA) was inserted into the right femoral vein and advanced to the right atrium.

### Experimental protocol (Fig. 4)

**Figure 4:**
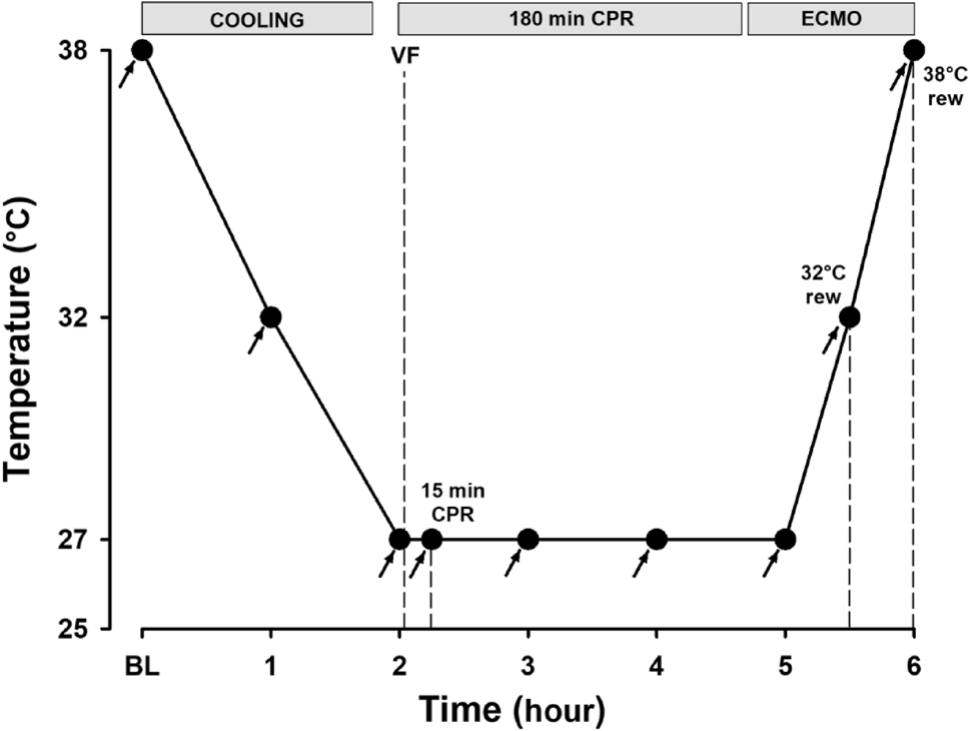
Experimental protocol. Arrows depict time points for hemodynamic measurements, blood sampling, and microsphere administration.

After immersion cooling in ice water to a blood-temperature of 27 °C, hypothermic cardiac arrest was induced by stimulating the epicardial surface with an alternating current (5–20 mA, 6 Hz, and 30 V). To achieve this, a 15 cm long needle electrode was inserted in the epigastric area and directed towards the apex of the heart guided by suctioning of blood from the left ventricle. Hypothermic cardiac arrest was defined as the appearance of ventricular fibrillation on the electrocardiogram and simultaneous absence of fluctuation in arterial pressure. After 90 s of hypothermic cardiac arrest, CPR was started using an automated chest compression device (LUCAS chest compression system, Physio-Control Inc., Lund, Sweden). Active decompression was achieved as the piston on this compression device was equipped with a suction cup to ensure a continuous compression/decompression mode with a duty cycle of 50 ± 5% at a frequency of 100 ± 5 compressions/min. Compression depth was 4–5 cm, and CPR was continued for 3 h. After 2.5 h of CPR, ECMO-instrumentation was started in order for all cannulas to be in placed at the 3-h mark. The animals were first rewarmed on ECMO (5 °C temperature gradient between core and arterial blood) to a blood temperature of 32 °C, and stabilized for 10 min, before blood sampling and recordings were performed. Electric cardioversion was attempted at 100 J up to three times. The animals were then rewarmed to a blood temperature of 38 °C (max heat exchanger temperature 38 °C), Thereafter, blood sampling and recordings were repeated and cardioversion was tried again. Based on the occurrence of multiple costae and sternal fractures made in our previous experiment after 3-h CPR^19^, if cardioversion was unsuccessful after 3 shocks, a sternotomy was made to evacuate extravascular blood, followed by internal defibrillation (5–15 J). The experiment was then concluded and the animals euthanized.

### Sampling

Mean arterial pressure (MAP), heart rate (HR), intracranial pressure (ICP), central venous pressure (CVP), and urinary output were recorded using PowerLAB 16/35 and LabChart software (ADInstruments, Dunedine, New Zealand). CO was measured by a thermodilution technique as described by Carretero and colleagues^54^, also during CPR, using 10 ml cold saline injected into the pulmonary artery catheter and recorded on a Vigilance monitor (Edwards Lifesciences, Irvine, CA, USA). During rewarming, ECMO pump flow rate was used as a surrogate for CO. All samplings were obtained at three core temperatures: baseline 38 °C, during cooling at 32° and at 27 °C. Samples were also obtained during CPR at 15, 60, 120 and 180 min, and during rewarming at 32° and 38 °C. At all sampling points, approximately 10 million stable isotope labelled 15 µm microspheres (BioPAL Inc., Worcester, MA, USA) were injected to determine organ blood flow. Simultaneously, a reference blood sample was drawn from the aortic arch at a constant rate (5 ml/min, 2 min). Differently labelled microspheres were used at all sampling points. During cooling and CPR, the microspheres were injected into the left ventricle through the pigtail catheter. During rewarming, the microspheres were injected through the injection port on the arterial ECMO cannula. After the experiment, tissue samples were collected and sent for neutron activation analysis (BioPAL Inc.) along with reference blood samples to measure sample radioactivity in disintegrations per minute (dpm). Organ blood flow was determined as already described in detail^53^. Briefly, organ blood flow (ml/min/g) was calculated as the product of tissue sample activity (dpm) and reference sample flow rate (ml/min), divided by the product of reference blood sample activity (dpm) and tissue sample weight (g).

### Calculations

Blood gases were analysed in arterial, central venous, and the jugular bulb samples using ABL800 FLEX (Radiometer medical, Copenhagen, Denmark). O_2_ content (ml/100 ml) values (arterial, central venous, and jugular bulb) was calculated according to the formula: SaO_2_ × Hb × (1.34 × 10^–2^) + 0.0031 × PO_2_ × 7.5, where SaO_2_ is blood O_2_ saturation (%), Hb is haemoglobin (g/dl) determined in venous blood, and PO_2_ is partial oxygen tension in blood (kPa). At baseline, during cooling and 3-h CPR, global ḊO_2_ was calculated as the product of CO and arterial O_2_ content per kg body weight (ml/min/kg). Global V̇O_2_ was calculated as the product of CO and the difference between arterial and venous O_2_ content per kg body weight (ml/min/kg). During rewarming, ECMO flow rate was used instead of CO to calculate global ḊO_2_ and V̇O_2_. Cerebral ḊO_2_ was calculated as the product of cerebral blood flow in ml/100 g brain tissue and arterial O_2_ content (ml/min/100 g). Cerebral V̇O_2_ was calculated as the product of cerebral blood flow in ml/100 g brain tissue and the difference between arterial and jugular bulb O_2_ content (ml/min/100 g). Cerebral blood flow values were calculated as mean of pooled data of left and right brain blood flow (temporal lobes and cerebellum). Global and cerebral O_2_ extraction rate was calculated as the ratio of corresponding V̇O_2_ to ḊO_2_ values. Cerebral perfusion pressure was calculated as a difference between MAP and ICP.

### Biochemistry

The selection of biomarkers to monitor organ function and to detect organ injury were based on a previous report^40^. Alanine aminotransferase (ALAT), aspartate aminotransferase (ASAT), alkaline phosphatase (ALP), amylase, total bilirubin, creatinine, lipase, urea, and γ-glutamyl transferase (γ GT) were analysed by a colorimetric method in plasma samples using a Cobas 8000 analyser (Roche Diagnostics GmbH, Mannheim, Germany). Porcine soluble protein-100β (s-100β), porcine adrenomedullin (ADM), porcine activin A, porcine neuron-specific enolase (NSE), porcine glial fibrillary acidic protein (GFAP), porcine ubiquitin carboxyl terminal hydrolase L1 (UCHL1), porcine creatine kinase MB isoenzyme (CK-MB), and porcine cardiac troponin T type 2 (cTn-T) were analysed in plasma samples using ELISA kits (MyBioSource Inc., San Diego, CA, USA, and Nordic BioSite AB, Täby, Sweden).

### Statistics

Statistical analysis was performed using Sigma Plot statistical software version 14 (Systat Software Inc. (SSI), Richmond, CA, USA). Normal distribution was assessed using the Shapiro–Wilk test. Intragroup comparisons were performed by one-way repeated measures ANOVA for normal distributed variables, and Friedman repeated measures ANOVA on ranks for non-normal distributed variables. If significant differences were found, Dunnett’s post hoc test was used to compare values within group vs. baseline. The level of significance was set at *p* < 0.05. Data are presented as means and SD.

## Data Availability

The datasets generated during and/or analysed during the current study are available from the corresponding author on reasonable request.
